# The effects of dietary and lifestyle interventions among pregnant women who are overweight or obese on longer-term maternal and early childhood outcomes: protocol for an individual participant data (IPD) meta-analysis

**DOI:** 10.1186/s13643-017-0442-6

**Published:** 2017-03-09

**Authors:** Jodie M. Dodd, Rosalie M. Grivell, Jennie Louise, Andrea R. Deussen, Lynne Giles, Ben W. Mol, Christina Vinter, Mette Tanvig, Dorte Moller Jensen, Annick Bogaerts, Roland Devlieger, Riitta Luoto, Fionnuala McAuliffe, Kristina Renault, Emma Carlsen, Nina Geiker, Lucilla Poston, Annette Briley, Shakila Thangaratinam, Ewelina Rogozinska, Julie A. Owens

**Affiliations:** 1 The University of Adelaide, Discipline of Obstetrics & Gynaecology, and Robinson Research Institute, Adelaide, South Australia Australia; 2 Department of Perinatal Medicine, Women’s and Children’s Hospital, 72 King William Road, North Adelaide, South Australia 5006 Australia; 3 The University of Adelaide, School of Public Health, Adelaide, South Australia Australia; 4 Institute of Clinical Research, University of Southern Denmark, 5230 Odense M, Denmark; 5 Department of Gynecology and Obstetrics, Odense University Hospital, Odense, Denmark; 6 Department of Endocrinology, Odense University Hospital, 5000 Odense C, Denmark; 7 Department of Healthcare Research, PHL University College, Limburg Catholic University College, Hasselt, Belgium; 8 Division of Mother and Child, Department of Obstetrics and Gynaecology, University Hospitals KU Leuven, Leuven, Belgium; 9 UKK Institute for Health Promotion, Tampere, Finland; 10 School of Medicine and Medical Science, UCD Institute of Food and Health, Dublin, Ireland; 11 Department of Obstetrics and Gynaecology, Hvidovre Hospital, University of Copenhagen, Hvidovre, Denmark; 12 Herlev and Gentofte Hospital Clinical Nutrition Research Unit, Copenhagen University Herlev, Herlev, Denmark; 13 Division of Women’s Health, Women’s Health Academic Centre, King’s College London, St. Thomas’ Hospital, London, UK; 14 Multidisciplinary Evidence Synthesis Hub (mEsh), Barts and The London School of Medicine and Dentistry, Queen Mary University of London, London, UK; 15 Women’s Health Research Unit, Barts and The London School of Medicine and Dentistry, Queen Mary University of London, London, UK

**Keywords:** Childhood obesity, Pregnancy lifestyle intervention, Individual participant data meta-analysis

## Abstract

**Background:**

The aim of this individual participant data meta-analysis (IPDMA) is to evaluate the effects of dietary and lifestyle interventions among pregnant women who are overweight or obese on later maternal and early childhood outcomes at ages 3–5 years.

**Methods/design:**

We will build on the established International Weight Management in Pregnancy (i-WIP) IPD Collaborative Network, having identified researchers who have conducted randomised dietary and lifestyle interventions among pregnant women who are overweight or obese, and where ongoing childhood follow-up of participants has been or is being undertaken. The primary maternal outcome is a diagnosis of maternal metabolic syndrome. The primary childhood outcome is BMI above 90%.

We have identified 7 relevant trials, involving 5425 women who were overweight or obese during pregnancy, with approximately 3544 women and children with follow-up assessments available for inclusion in the meta-analysis.

**Discussion:**

The proposed IPDMA provides an opportunity to evaluate the effect of dietary and lifestyle interventions among pregnant women who are overweight or obese on later maternal and early childhood health outcomes, including risk of obesity. This knowledge is essential to effectively translate research findings into clinical practice and public health policy.

**Systematic review registration:**

This IPD has been prospectively registered (PROSPERO), ID number CRD42016047165.

**Electronic supplementary material:**

The online version of this article (doi:10.1186/s13643-017-0442-6) contains supplementary material, which is available to authorized users.

## Background

Globally, 1.46 billion adults [1], and 170 million children under the age of 18 years [2], are estimated to be overweight or obese. Obesity is occurring at an increasingly early age, affecting more than 43 million children aged 0–5 years world-wide [3], leading the World Health Organization to describe childhood obesity as “one of the most serious public health challenges of the 21st century [4]”. With obesity occurring earlier in life, the aggregate exposure and risk of deleterious health consequences also increases [5].

A variety of factors have been identified as increasing an individual’s risk of increased adiposity and obesity in childhood. In particular, both high maternal BMI and excessive gestational weight gain during pregnancy have been consistently identified as significant independent pre-natal factors [6–9]. Recognized postnatal factors operating in early infancy include feeding (exposure to breast feeding, formula feeding, and introduction of solids), activity (including sedentary behaviours), and sleep duration, although the associations between parental and postnatal factors are complex, and the extent to which they may be modified by intrauterine events is unclear [10].

Maternal overweight and obesity represents a significant health issue for women during pregnancy and childbirth, with estimates suggesting that over 50% of women enter pregnancy with a BMI in excess of 25 kg/m^2^ [11]. While there are well-documented risks associated with obesity during pregnancy for both the woman and her infant [12, 13], there is an increasing recognition of the association between maternal obesity and subsequent obesity in her offspring, developing in early infancy [14] and extending into later childhood [15–17]. Independent of maternal obesity, high gestational weight gain (GWG) is also associated with an increased risk of pre-school obesity [17–19].

Excessive GWG is recognized as a significant risk factor for maternal postpartum weight retention and the subsequent development of obesity [20–22], contributing to an increase in inter-pregnancy BMI, and an increased risk of adverse outcomes in a subsequent pregnancy [22–24]. Excessive GWG is also consistently associated with a greatly increased risk of a woman subsequently developing diabetes in later life [25, 26], when compared with women whose weight gain was within the IoM recommendations. A similar increase in risk of cardiovascular disease in later life has also been reported among women with excessive pregnancy weight gain [27–30].

The major complications associated with obesity during pregnancy and childbirth for women and their infants are well defined, and a comprehensive systematic review led by Thangaratinam and colleagues [31] has identified numerous studies assessing dietary and lifestyle interventions in pregnancy. However, the effect of these pregnancy interventions on later maternal and early childhood health is yet to be evaluated. The International Weight Management in Pregnancy (i-WIP) Collaborative group is near completion of an individual participant data meta-analysis (IPDMA), funded by the UK NIHR, evaluating the effect of dietary and lifestyle interventions during pregnancy on short-term pregnancy and birth outcomes [32]. We propose to extend this existing collaboration to evaluate the effect of dietary and lifestyle interventions among pregnant women who are overweight or obese on later maternal and early childhood health outcomes, establishing the International Weight Management in Pregnancy Collaboration: 3-year follow-up (i-WIP-3).

While systematic review and traditional methods of meta-analysis generate robust evidence regarding healthcare interventions [33], recent calls have advocated that level-1 evidence be attributed to IPDMA [34, 35]. Despite this, IPDMA remains under-utilised as a methodological tool, limiting the quality and robustness of clinical care guidelines and their recommendations [35]. Use of IPDMA methodology greatly increases statistical power to generate unequivocal pooled risk estimates and to identify key maternal characteristics and critical components of the intervention contributing to maternal and childhood health. Combining and analyzing the extensive volume of randomised controlled trial (RCT) data available world-wide avoids the expense, duplication of effort, and inevitable time delays of undertaking further very large-scale trial(s) with pre-specified longer-term primary outcomes relating to maternal and child cardio-metabolic health.

## Aims

Using IPDMA, we will determine the effects of antenatal dietary and lifestyle interventions in pregnancy for women who are overweight or obese on longer-term health outcomes for the women and their children at 3-5 years of age; this will also be assessed in particular groups at greater risk to inform potential targeting of the intervention.

With the available raw participant level data, we will address the effect of antenatal dietary and lifestyle interventions on longer-term health outcomes, overall, and according to maternal subgroups. The primary subgroup analysis is early pregnancy body mass index (BMI) category (25.0–29.9 vs ≥30.0 kg/m^2^). Secondary subgroup analyses will also be carried out on subgroups defined by the following characteristics:

1. Ethnicity (Caucasian vs Asian vs African);
2. Socioeconomic status (high vs low social disadvantage) at time of randomisation during pregnancy;
3. Parity (0 vs ≥1) at time of randomisation during pregnancy; and
4. Gestational weight gain (as a continuous variable, and according to adherence to the Institute of Medicine (IoM) gestational weight gain recommendations) [36].

## Methods

### Study design

We will conduct an IPDMA, utilising an approach that follows existing guidelines and that complies with the PRISMA-IPD statement and recent reporting guidelines for IPD meta-analysis (Additional file 1) [37].

### Inclusion criteria for the studies and search strategy

Individual patient data from RCTs in which women with a singleton, live gestation between 10^+0^–20^+0^ weeks, and of BMI ≥ 25.0 kg/m^2^ at the time of the first antenatal visit, were randomised to receive a diet and/or lifestyle intervention or continued standard antenatal care, and in which longer-term maternal and child follow-up has been undertaken. The current studies were identified by a systematic literature search within the i-WIP collaboration [32]. We will update our previously described literature search, without language restrictions, in order to identify any new potential relevant trials with planned or published follow-up at the commencement of the project and 1 year prior to its completion to minimise the potential to miss relevant trials [32].

To date, we have identified seven such randomised trials, with collaborators providing in principal agreement to access de-identified individual participant data for women and their children. The characteristics of each trial are presented in Table 1. We are aware of two studies currently recruiting to the primary pregnancy intervention, with an intention to conduct longer-term maternal and child follow-up [38, 39].

**Table 1:** Characteristics of the identified randomised trials

Trial name	LIMIT [55–57]	UPBEAT [58]	ROLO [59]	LiP [60–63]	Belgian Flanders [64]	NELLI [65]	The TOP study [66]
Investigators	Dodd, Grivell, Owens	Poston	McAuliffe	Vinter, Tanvig, Jensen	Bogaerts, Devlieger	Luoto	Renault
Funding	NHMRC (ID 519240 and 1043178)	NIHR	HRB Ireland	Trygfonden, Denmark		Finnish Diabetes Research Fund	Sygekassernes Helsefond; Broedrene Hartmann Fondon
Inclusion criteria	Overweight or obese	Obese	Women with prior birth of infant >4 kg	Obese	Obese	Women of all BMI categories	Obese
Setting	Adelaide, South Australia	Multiple centres, United Kingdom	Dublin, Ireland	Odense, Denmark	Multiple centres, Belgium	Pirkananaa region, Southwest Finland	Copenhagen, Denmark
Intervention intensity	3 face-to-face sessions (2 with dietitian, 1 with RA); 3 telephone contacts	8 weekly sessions with health trainer	Initial group dietary education session, 2 follow-up sessions with dietitian	4 sessions with dietitian; weekly physiotherapy sessions	4 sessions with midwife	5 counselling sessions with midwife	Up to 13 sessions (alternate face-to-face and telephone with dietitian)
Intervention content	Healthy eating in pregnancy; food substitutions; promote increased physical activity; identify barriers/enablers	Healthy eating; food substitutions; promote increased physical activity; SMART goals	Healthy eating in pregnancy; low glycaemic index foods	Dietary advice consistent with national guidelines; individual energy requirements calculated; moderate physical activity	Dietary advice consistent with national guidelines	Promote increased physical activity; dietary advice consistent with national guidelines	Mediterranean style diet consistent with national recommendations; increased physical activity measured by pedometer
Baseline data	10–20 weeks gestation	15–19 weeks gestation	Prior to 18 weeks gestation	10–14 weeks gestation	Prior to 15 weeks gestation	8–12 weeks gestation	Prior to 16 weeks gestation
Primary trial findings	Reduction infant birth weight >4 kg Improved diet and physical activity	Reduction GWG 0.5 kg Improved diet and physical activity	Reduction GWG 1.3 kg Improved diet, physical activity and insulin resistance	Reduction GWG 1.6 kg Improved insulin resistance	Reduction GWG 1.1 kg	Reduction infant birth weight >4 kg Improved diet and physical activity	Reduction GWG 1.38 kg
Sample size	2212 women randomised	1556 women randomised	429 women randomised	360 women randomised	205 women randomised	238 women randomised	425 women randomised
Follow-up	6, 18, and 36 months	6 and 36 months	6 and 60 months	6 and 36 months	36 months	36 months	6, 9, 18, and 36 months
Retention	72% (~1592)	60% (~930)	68% (~290)	54% (~194)	65% (~134)	65% (~154)	65% (~250)

### Types of participants

Women with a singleton, live gestation between 10^+0^–20^+0^ weeks and of BMI ≥ 25.0 kg/m^2^ at the time of their first antenatal visit, and in which subsequent maternal and child follow-up has been planned or undertaken at 3–5 years of age.

### Types of interventions

Antenatal dietary and/or lifestyle interventions compared with continued standard antenatal care.

### Data collection and management

Each trial will contribute de-identified patient level data for each participant randomised. This will be stored in a secure, centralised database with access available only to authorised members of the i-WIP-3 data management group. The database will build on that currently used in the i-WIP collaboration. Specifically, data already housed has been coded for anonymity and relates to the women randomised (date of birth, center identification); baseline descriptive information (age, parity, ethnicity, BMI, smoking status, socioeconomic status, expected date of confinement); allocated treatment intervention; and pregnancy and birth outcome variables.

All data provided from individual trials will be checked to ensure internal consistency as well as consistency with published reports and to assess missing data, using published data, trial protocols, and data collection sheets. Similarly, the randomisation process for each trial will be checked, including the chronological randomisation sequence, stratification variables, and allocation assignment to consider the distribution of prognostic factors across treatment groups. Where inconsistencies are identified, they will be discussed with the individual investigators and will be resolved through consensus. In the initial stages, each trial will be analysed separately, and the output generated cross-checked against published data and verified by the individual investigator before being incorporated into the combined database.

### Variables available through the i-WIP collaboration

Trial level information has already been collected and exists within the database. This includes the number of women randomised; methods of random allocation; stratification variables; methods of allocation concealment; blinding of outcome assessment; nature of the intervention (including the content of the intervention, and the number and format in which the sessions were provided); nature of standard antenatal care provided.

Maternal participant level information has already been collected and exists within the database. This includes a unique participant code to ensure anonymity; maternal age; body mass index; parity; ethnicity; smoking status; and socioeconomic status.

Maternal pregnancy and birth outcome variables occurring after the time of randomisation have been collected and exist within the database. This includes pregnancy outcomes (including gestational diabetes, pre-eclampsia, and hypertension, pre-term birth, gestational weight gain); and birth outcomes (including induction of labour, mode of birth, estimated blood loss). Maternal dietary and physical activity reports are also available.

Neonatal participant level information has already been collected and exists within the database. A unique neonatal identification code will be linked to the maternal identification code to ensure anonymity. Outcome variables include gestational age at birth, birth weight, small and large for gestational age, perinatal death, shoulder dystocia, birth trauma, and admission to the neonatal intensive care unit (NICU).

### Variables to be collected through the i-WIP-3 Collaboration

Extended maternal participant level information will be collected and entered into an expanded database housing the above information. Outcome variables will include height, weight, BMI, skinfold thickness measurements, calculated percentage body fat and fat-free mass, dietary and physical activity patterns, blood pressure, quality of life and emotional wellbeing, and general health (Table 2).

**Table 2:** Available maternal health outcomes

Trial name	LIMIT [55–57]	UPBEAT [58]	ROLO [59]	LiP [60–63]	Belgian Flanders [64]	NELLI [65]	The TOP study [66]
Investigators	Dodd, Grivell, Owens	Poston	McAuliffe	Vinter, Tanvig, Jensen	Bogaerts, Devlieger	Luoto	Renault
Timing assessment	3 years	3 years	5 years		4–7 years	5–7 years	3 years
Height	✓	✓	✓	✓	✓	✓	✓
Weight	✓	✓	✓	✓	✓	✓	✓
Weight change	✓	✓	✓	✓	✓	✓	✓
BMI	✓	✓	✓	✓	✓	✓	✓
Circumferences							
-Neck		✓					
-Waist		✓			✓		
-Hip		✓					
-Thigh		✓					
-Mid upper arm		✓					
-Wrist		✓					
SFTM							
-Subscapular		✓	✓				
-Triceps		✓	✓				
-Biceps		✓	✓				
-Suprailiac		✓	✓				
% Body fat		✓	✓				
DXA			✓ Subset				
Bodpod		✓					
BIA		✓	✓				
Dietary intake	✓ Also 6 months, 18 months	✓			✓	✓	
Physical activity	✓ Also 6 months, 18 months	✓			✓	✓	
Metabolic syndrome		✓	✓			✓	
Glucose intolerance	✓ Subset	✓	✓			✓	At 12 months postpartum
Insulin						✓	
Type 2 DM	✓ Subset	✓	✓ Self report	✓ Fasting glucose		✓	
Hypertension/BP	✓	✓	✓		✓	✓	✓
Hyperlipidaemia		✓	✓			✓	
Inflammatory markers			✓			✓	
Transferrin???						✓	
DNA			✓ Saliva			✓	
RNA						✓	
Anxiety	✓ Also 6 months, 18 months					✓	
Anxiety-eating			✓ TFEQ			✓	
Depression	✓ Also 6 months, 18 months	✓	✓			✓	
Quality of life	✓ Also 6 months, 18 months	✓	✓			✓	
Health	✓	✓			✓	✓	
Reproductive health history	✓	✓	✓		✓	✓	
Life events					✓	✓	

Childhood participant level information will be collected and entered into an expanded database housing the above information. A unique childhood identification code will be linked to the neonatal and maternal identification codes to ensure anonymity. Outcome variables will include height, weight, BMI, skinfold thickness measurements, calculated percentage body fat and fat-free mass, dietary and physical activity patterns, blood pressure, neurodevelopmental outcome domains, and general health (Table 3).

**Table 3:** Available child outcomes

Trial name	LIMIT [55–57]	UPBEAT [58]	ROLO [59]	LiP [60–63]	Belgian Flanders [64]	NELLI [65]	The TOP study [66]
Investigators	Dodd, Grivell, Owens	Poston	McAuliffe	Vinter, Tanvig, Jensen	Bogaerts, Devlieger	Luoto	Renault
Timing assessment	6 months, 18 months, 3 years	6 months, 3 years	6 months, 5 years	6 months, 3 years	6 months, 3 years	6 months, 3 years	6 months, 9 months, 18 months, 3 years
Height	✓	✓	✓	✓	✓	✓	✓
Weight	✓	✓	✓	✓	✓	✓	✓
BMI	✓	✓	✓	✓	✓	✓	✓
Head circumference	✓	✓	✓				✓
Neck circumference			✓				✓
Arm circumference	✓	✓	✓				✓
Chest circumference	✓		✓				
Abdominal circumference	✓	✓	✓	✓	✓		✓
Thigh circumference			✓		✓		
Hip circumference		✓	✓	✓	✓		
Biceps SFTM	✓	✓	✓		✓		
Triceps SFTM	✓	✓	✓	✓	✓		
Subscapular SFTM	✓		✓	✓	✓		
Suprailiac SFTM	✓				✓		
Abdominal SFTM	✓						
Thigh SFTM	✓	✓	✓				
Fat mass (calculated)	✓	✓	✓	✓		✓	✓
DEXA			✓	✓			✓
BOD-POD		✓	✓				
Blood pressure	✓	✓	✓	✓	✓		
Cardiovascular function		✓	✓				
Breast feeding	✓	✓	✓	✓	✓	✓	✓
Dietary patterns	✓	✓	✓	✓	✓		
Physical activity	✓	✓	✓		✓	✓	✓
Sedentary behaviours	✓	✓	✓	✓	✓		
Sleep time	✓	✓	✓				✓
General health	✓	✓	✓	✓		✓	✓
Ages and stages	✓	✓	✓		✓		✓
Cognitive development	✓	✓	✓		✓		✓
Smoking environment	✓	✓	✓	✓	✓		✓
Blood specimen		✓	✓	✓			✓
Salivary specimen	✓	✓				✓	

### Establishment of outcome measures

To determine primary and secondary maternal and child outcomes, we conducted a Delphi survey. An initial list of outcomes was incorporated into a previous Delphi survey conducted by the i-WIP group, with a specific focus on outcomes relevant to women and their children [40]. We undertook a two-stage Delphi survey (February to April 2016) using a methodology consistent with current recommendations [41], in order to prioritise maternal and childhood outcomes of relevance to clinical practice. The panel involved members of the i-WIP collaborative steering committee, members from the planned IPD investigators, and other identified multi-disciplinary experts in the field.

An online survey tool was used and a link sent to individual members of the panel, who were asked to score each listed maternal and childhood outcome in terms of clinical relevance for patient care. A nine-point Likert scale was used to evaluate the importance, with a score of 9 considered critical, while a score of 1 was considered of limited importance to patient care. In the first round of the survey, participants were asked to suggest other relevant outcomes that may not have been included. These additional outcomes were included in the second round of the survey. For each outcome, a median and interquartile range (IQR) was calculated. This information was then provided to panelists in the second round of the survey. For each survey round, an e-mail reminder was sent if no response had been received within 2 weeks, with a second reminder sent after 4 weeks.

A total of 21 of 41 individuals completed both the first and second rounds of the survey. The median and IQR for maternal and childhood outcomes are shown in Tables 4 and 5.

**Table 4:** Maternal outcomes determined from the Delphi survey (first round median and IQR in brackets)

Maternal outcomes	Median	IQR
Maternal metabolic syndrome (8.00, 2.00)	8	1
Maternal glucose intolerance (8.00, 2.00)	8	1.25
Maternal type 2 diabetes (8.00, 2.00)	8	2
Maternal hypertension (8.00, 2.00)	7.5	1
Maternal physical activity patterns (6.00, 3.00)	7	1
Maternal weight change (7.00, 3.50)	7	1.25
Maternal hyperlipidaemia (6.00, 3.00)	6	2
Maternal dietary patterns (6.00, 3.50)	6	2
Maternal anxiety/depression (6.00, 2.00)	6	2
Maternal quality of life (6.00, 2.00)	6	1.25
Maternal body fat (%) (5.00, 2.00)	5	2
Maternal elevated CRP (4.00, 3.00)	4.5	2
Maternal skin fold thicknesses (5.00, 2.00)	4.5	2

**Table 5:** Childhood outcomes determined from the Delphi survey (first round median and IQR in brackets)

Child outcomes	Median	IQR
BMI (7.00, 2.50)	7	2
BMI ≥ 85% (7.00, 1.50)	7	1.25
BMI *z* score (7.00, 2.00)	7	1.25
Blood pressure-continuous (7.00, 2.00)	7	1.25
Hypertension (SBP and DBP) (7.00, 2.00)	7	1.25
Abdominal fat mass (7.00, 3.00)	7	1.25
Impaired cardiovascular function (8.00, 2.50)	7	2.25
Physical activity measures/patterns (7.00, 2.50)	7	2.25
Sedentary activity measures (7.00, 3.00)	7	2
Food frequency/eating–FFQ (7.00, 3.00)	7	1.25
Developmental milestones (7.00, 2.50)	7	2
General health (7.00, 1.50)	7	1
Insulin/glucose homeostasis (7.00, 2.50)	7	2
Cognitive development (new)	7	2.25
Lipid profile (7.00, 2.50)	6.5	1.25
Circumferences (6.00, 2.00)	6	1
Skinfold thicknesses (6.00, 1.00)	6	1
Calculated fat-free mass (%) (6.00, 1.50)	6	1.25
Calculated total fat mass (%) (6.00, 1.50)	6	1.25
Breastfeeding duration (6.00, 2.00)	6	1.25
Sleep patterns/duration (6.00, 2.00)	6	1
Leptin/adiponectin (6.00, 2.00)	6	1
Asthma/allergy (new)	6	1
Child behaviour (new)	6	2
Inflammatory markers (6.00, 2.00)	5	2

### Primary outcome measures

The primary maternal outcome is a diagnosis of maternal metabolic syndrome. The primary childhood outcome is BMI above 90%. Secondary outcome measures are presented in Tables 2 and 3.

### Data management and statistical analyses

#### Data checking

The IPDMA project coordinator will check all new maternal and child follow-up data provided from the individual trials to ensure internal consistency and consistency with published reports and to assess missing data, using published data, trial protocols, and data collection sheets. Similarly, the randomisation process for each trial will be checked, including the chronological randomisation sequence, stratification variables, and allocation assignment to consider the distribution of prognostic factors across treatment groups. Where inconsistencies are identified, they will be discussed with the individual investigators and resolved through consensus. Initially, each trial will be analysed separately, and the output generated will be cross-checked against published data and verified by the individual investigator before being incorporated into the IPDMA database.

#### Data transformation

Data from each trial will be combined in a common data set, after the above. Each patient will retain a unique trial identifier. The combined data set will be used to define the new variables required to address the hypotheses of the proposal.

#### Statistical analyses

A detailed statistical analysis plan will be prepared and agreed upon by members of the i-WIP-3 collaboration, prior to the conduct of any analyses. All randomised participants with available outcome data will be included in the analyses, on an intention-to-treat basis, according to the treatment group to which the woman was allocated at the time of randomisation (dietary and/or lifestyle intervention vs standard care). Imputation methods will be used to account for missing data.

The statistical analyses of this IPDMA will utilize methods described in the Cochrane Collaboration Handbook [42] and as outlined for the i-WIP IPDMA [32]. Firstly, we will summarise the overall effect of each intervention in relation to each outcome. Meta-analyses of the effectiveness of antenatal interventions in pregnancy will be performed for the primary and secondary maternal and child outcomes. We will include all patients randomised and will adopt intention-to-treat principles.

All trials will be reanalysed separately, and the investigators asked to confirm their individual results, with resolution of any discrepancies. We will then perform a one-step IPDMA to generate a pooled intervention effect. A two-step IPDMA will also be conducted as a secondary analysis for comparison with the one-step results, unless convergence issues with the one-step analysis require the use of the two-step approach. A one-step approach accounts for clustering of participants within studies and analyses IPD from all trials simultaneously. A two-step approach first estimates the intervention effect from the IPD in each study separately and then pools them using a conventional meta-analysis of the intervention effect estimates obtained. While one and two-step meta-analyses typically yield similar results, both will be undertaken to ensure robust conclusions [43, 44].

We anticipate that there will be evidence of heterogeneity in our IPDMA and will therefore use a random effects approach to account for between study heterogeneity in intervention effect. Heterogeneity will be summarised using the I-squared statistic (which describes the proportion of total variability due to between study heterogeneity) and the estimated between-study variance (‘tau-squared’).

For continuous outcomes, we will use mean differences, which will be standardized where possible if outcome scales differ substantially, with adjustment for baseline values using analysis of covariance [45]. For binary outcomes, we will calculate relative risks and incorporate modelling where required (for example, logistic regression to adjust for clustering). We will assume that the random effects contributing to heterogeneity at the individual trial level will be normally distributed, although it is unlikely that violation of this assumption will affect the results of the analysis [46].

The subgroups to be considered as causes of heterogeneity and potential modifiers of the effect of the intervention include maternal BMI category, ethnicity, socioeconomic status, parity, and maternal gestational weight gain. We will generate summary intervention effects in each subgroup using the same random-effects meta-analysis approach as described above. Subgroup analyses, if not carefully planned, can lead to misleading results, and we will therefore exercise caution in the interpretation of subgroup results, with adjustment for multiple testing.

To explore the possibility of chance effects contributing to the findings of our subgroup analyses, we will incorporate treatment-covariate interaction terms in the analysis. This will ensure that we estimate the pooled within-trial interaction of interest separately from the across-trial (meta-regression) interaction, as recommended because the former is the desired information as it is based solely on patient-level information [47, 48].

A secondary analysis will evaluate the association between maternal gestational weight gain and maternal and child outcomes in overweight and obese women. We will fit a suitable regression model to account for clustering of participants within individual trials and quantify how each 1-unit increase in weight gain changes the risk of each outcome. As the relationship may be non-linear, we will use fractional polynomial terms [49]. Modelling will use linear regression for continuous variables and logistic regression for binary variables, and will also account for clustering of participants within trials, as well as their allocated treatment intervention during pregnancy.

### Study quality assessment and evaluation of nonresponse bias

We will consider all recorded variables, even those not reported in the published studies. The quality of each trial will be assessed [50, 51] to evaluate the integrity of the randomisation and follow-up procedure. We will evaluate the risk of bias in individual studies by considering six items used in the Cochrane risk of bias tool: sequence generation, allocation concealment, blinding, incomplete outcome data, selective outcome reporting, and other potential sources of bias.

If individual patient data are not received from all identified studies, the potential for bias due to nonresponse will be assessed by descriptive comparison of participating and non-participating studies, in terms of study characteristics (sample size, where and when conducted, etc.), patient population, and main effect estimate (where possible). Comparison of results from the two-stage meta-analysis with those of the one-stage analysis will also be used to assess the potential for nonresponse bias.

### Sample size

There are a total of 3544 women and children with data available at the 3-year follow-up across the 7 trials; the smallest study has a sample size of 134. Power and coverage (alpha/type I error) for the main child outcome (BMI > 90%) and for the main subgroup analysis (interaction between treatment and maternal early pregnancy BMI category) were investigated across 1000 simulated datasets using the ipdpower command in Stata [52]. The simulated datasets incorporated study-specific fixed intercepts (base log-odds), fixed effects for maternal BMI category and treatment-by-BMI interaction, and random effects for treatment.

For the main childhood outcome (BMI > 90%), assuming a reduction in incidence from 21.0% in the control group to 16.7% in the treatment group (corresponding to a treatment effect OR of approximately 0.75), and stipulating a high degree of between-study heterogeneity for the random treatment effect (SD on the log-odds scale corresponding to 1/3 of the average effect), there was 85% power (95% CI 82.6–87.2%) to detect the treatment effect. Coverage was acceptable at 92% (95% CI 90.1–93.6).

For the main subgroup analysis, assuming that the treatment effect in the higher BMI category is approximately 1.5 times that in the lower BMI category (OR of 1.5); that the control group OR for the higher BMI category compared to the lower BMI category is approximately 1.6, and that about 70% of women have early pregnancy BMI in the higher category (≥30.0), there is greater than 80% power to detect the interaction effect (82.2%, with 95% CI 79.6–84.5%), with good coverage of 96.4% (95% CI 95.0–97.5%).

### Management considerations

The i-WIP-3 Collaboration will have a steering group consisting of the current named authors, which includes a representative from each of the individual randomised trials contributing individual patient data, in addition to the project coordinator and statistician. The steering committee will meet initially to discuss and finalise the definitions and outcomes to be assessed and the statistical processes proposed. Where possible, one face-to-face collaborator meeting will be scheduled each year, at which key decisions, including the project design, analysis plan, and interpretation of findings will be discussed.

The operational requirements of the project will be performed by the project coordinator and statistician, in conjunction with the individual trial managers and statisticians and will be overseen by the chair of the steering committee.

### Publication considerations

Each member of the steering committee will be provided with the results of the analysis, and a meeting will be held to discuss and interpret the findings. The current named authors will be responsible for the preparation of manuscripts, which will then be circulated to each member of the committee for further discussion prior to submission for publication. Where possible, each member of the steering committee will be named as an author on any publications arising from the analysis and on behalf of the i-WIP-3 collaboration as a whole, with acknowledgement of all participating collaborators within the manuscript.

## Discussion

There is an increasing recognition of the association between maternal obesity, high infant birth weight, and the subsequent development of childhood obesity. In a large population cohort from the United States, the overall incidence of infant birth weight above 4 kg was approximately 12% [53]. However, approximately 1 in 5 children who were obese at ages 5–6 years had birth weight above 4 kg, increasing to almost one third of obese individuals at age 14 years [53]. Therefore, antenatal interventions which are successful in reducing the risk of maternal gestational weight and adiposity gain and/or high birth weight infants represent a public health strategy of considerable significance to tackle the global issue of increasing obesity and adverse health in children and in adults [54].

Our proposed IPDMA provides a unique opportunity to evaluate the effect of dietary and lifestyle interventions among pregnant women who are overweight or obese on later maternal and early childhood health outcomes, including risk of obesity. Importantly, we will build on the successful existing i-WIP project, which has standardized the baseline characteristics of women recruited to randomised trials, interventions, and short-term pregnancy and birth outcomes. While each of the identified randomised trials are sufficiently similar in design and outcomes to allow meaningful meta-analysis to occur, the intensity of the intervention provided and social demographics of included participants is diverse with the IPD proposed enabling identification of effect modifiers through pre-specified subgroup analyses.

Furthermore, this can be achieved in a relatively efficient manner and with sufficient statistical power, avoiding the expense, duplication of effort, and inevitable time delays in undertaking another large-scale pregnancy intervention trial with a pre-specified primary outcome of later maternal or early childhood obesity. This knowledge is essential to effectively translate research findings into clinical practice and public health policy, and to maximise the return on publicly funded research investments globally.

## Additional file

Additional file 1: PRISMA-P 2015 Checklist. (DOCX 30 kb)

## Abbreviations

BMI: Body mass index
GWG: Gestational weight gain
IPD: Individual participant data
IPDMA: Individual participant data meta-analysis
IQR: Interquartile range
i-WIP: International Weight Management in Pregnancy
NICU: Neonatal intensive care unit
OR: Odds ratio
RCT: Randomised controlled trial
UK-NIHR: United Kingdom-National Institute for Health Research
